# Leptin, vitamin D, and APRI values during the six months post-liver transplantation

**DOI:** 10.1186/s12876-026-04640-9

**Published:** 2026-02-20

**Authors:** Yasmine A. Algobashy, A. S. shehatta, Mabdel Wahab, Magdy M. Youssef

**Affiliations:** 1 Department of Biochemistry, Faculty of Science, Mansoura University, Mansoura, Egypt; 2 Gastrointestinal Surgery Center, Faculty of Medicine, Mansoura University, Mansoura, Egypt; 3 Department of Surgery, Faculty of Medicine, Gastrointestinal Surgery Center, Mansoura University, Mansoura, Egypt

**Keywords:** Liver fibrosis, Leptin, Vitamin D, APRI, FIB-4

## Abstract

**Background:**

Liver fibrosis is a common outcome of chronic liver disease, characterized by accumulating extracellular matrix proteins. Aims: While the gold standard in the assessment of liver fibrosis remains liver biopsy, non-invasive methods have been increasingly used for estimating liver fibrosis. This study aims at contributing to our understanding of the role of leptin,vitamin D, APRI, and FIB-4 in the progression of liver disease. For this purpose, we evaluate the clinical significance of leptin, vitamin D, APRI, and FIB-4 in cirrhotic patients with different ratios of steatosis and in cirrhotic pateints with different grades of hepatocellular carcinoma. We compare the performance of simple biochemical scores (FIB-4 and APRI) with leptin and vitamin D. Also, We identified the proteins in the serum of liver fibrosis and showed the role of these proteins in the pathogenesis of liver fibrosis. Also, we identified the relationship between leptin and vitamin D with extracellular matrix proteins using bioinformatics analysis.

**Methods:**

The current study included 50 liver fibrosis patients who underwent liver transplantation. We measured the levels of serum vitamin D and leptin in these patients at pre-LT and three- and six-months post-LT using enzyme-linked immunosorbent assay. We performed qualitative proteomic analysis to identify the proteins in the sera from patients with liver fibrosis. Among these proteins, there are extracellular matrix proteins. One-way ANOVA test was used.

**Results:**

We found that serum leptin levels and APRI values were significantly higher in liver fibrosis patients than in controls, after three- and six-months following LT serum leptin levels and APRI values significantly decreased. While serum vitamin D levels were significantly lower in liver fibrosis patients than in controls, after three- and six-months following LT, serum vitamin D levels significantly increased. ROC curves analysis showed that the area under the curve (AUC) of vitamin D and leptin was 1 and 0.997, respectively. Gene ontology analysis demonstrated that the proteins participate in a diverse array of biological processes, emphasizing the systemic effects of liver fibrosis disease. From the STRING database, the proteins are at least partially biologically connected, as a group with leptin and vitamin D explaining the promiscuous nature of leptin and vitamin D. This emphasized the role of leptin and vitamin D in accumulation and increased synthesis of extracellular matrix proteins that incorporated in the pathogenesis of liver fibrosis, and showed that the leptin and vitamin D are involved in the pathogenesis process where the total proteins are involved.

**Conclusions:**

In summary, serum vitamin D and leptin could serve as a valuable supplemental biomarker for predicting the liver fibrosis.

## Background

A significant global health concern is liver fibrosis brought on either viral or metabolic chronic liver disorders. Fibrosis is an important component that correlates with the course of liver disease and increases the risk of hepatocellular carcinoma in the liver disease [1].

Hepatotoxic and cholestatic liver injuries are the two types of chronic liver injury that lead to liver fibrosis. Cellular damage from external sources, such as viral infections causing hepatitis B and C and alcoholic and non-alcoholic steatohepatitis, can cause hepatotoxic injury. Primary biliary cholangitis, primary sclerosing cholangitis, and biliary atresia are among the primary (and secondary) diseases that induce cholestatic damage, which is defined by decreased or obstructed bile flow in the liver [2].

The primary cells in the wounded liver that produce collagen are activated hepatic stellate cells, portal fibroblasts, and myofibroblasts originating from bone marrow. Fibrogenic cytokines like TGF-β1, angiotensin II, and leptin stimulate these cells [3].

In 1994, Friedman and associates discovered leptin as the result of the obesity gene [4]. The central nervous system is alerted to the existence of adequate energy reserves by circulating leptin levels, which are correlated with adipose tissue mass. This reaction is typified by anorexia and increased energy expenditure [5]. Leptin regulates a wide range of functions, including wound healing, angiogenesis, insulin secretion, and the immune system [6]. Adipose tissue and HSC both express leptin [7]. Hepatic stellate cells (HSC) that are activated but not quiescent express leptin [8]. Fibrogenesis and the repair of liver wounds depend on the HSC activation mechanism [7]. As the quiescent phenotype of normal liver gives way to the activated state of the injured liver, these cells become receptive to various cytokines, can express various soluble mediators, and produce extracellular matrix components that ultimately contribute to the progression of fibrosis [9]. Hepatic stellate cells, the liver's principal effector cells, mediate leptin's profibrotic effect [10].

APRI, or preoperative aspartate aminotransferase to platelet count ratio index, has been found to be a biochemical marker for fibrosis and histology fibrogenesis in cirrhosis and poor prognosis of HCC [11]. Fibrosis-4 (FIB-4) is a scoring method that uses the patient's age, platelet count, aspartate transaminase (AST), and alanine transaminase (ALT) to determine the degree of liver fibrosis. In addition to being inexpensive, all of these factors are easily accessible to primary care physicians [4].

While the gold standard in the assessment of liver fibrosis remains liver biopsy, non-invasive methods have been increasingly used for estimating liver fibrosis. Present study aims at contributing to our understanding of the role of leptin,vitamin D, APRI, and FIB-4 in the progression of liver disease. For this purpose, we evaluate the clinical significance of leptin, vitamin D, APRI, and FIB-4 in cirrhotic patients with different ratios of steatosis and in cirrhotic pateints with different grades of hepatocellular carcinoma. We compare the performance of simple biochemical scores (FIB-4 and APRI) with leptin and vitamin D. Also, We identified the proteins in the serum of liver fibrosis and showed the role of these proteins in the pathogenesis of liver fibrosis. Also, we identified the relationship between leptin and vitamin D with extracellular matrix proteins using bioinformatics analysis..

## Materials and methods

This is a non-profit interventional study that was conducted on healthy subjects and patients with HCC and/or liver cirrhosis of various etiologies.. The pateints were diagnosed using liver biopsy. The subjects under examination underwent peripheral blood sampling to measure circulating levels of leptin and vitamin D as biomarkers of liver fibrosis.The initial blood draw (an 8 mL tube of serum) was taken on the day of hospital admission for all recruited cirrhotic and HCC participants. Following a diagnosis, each participant underwent routine outpatient clinical monitoring.. The second blood sample was taken from pateints at three and six months after liver transplantation.

The inclusion criteria were as follows: pateints who gave written informed consent; patients aged 18 years or older; patients with liver cirrhosis and/or hepatocellular carcinoma.

The exclusion criteria were as follows: pregnant women; pateints with gastrointestinal bleeding; patients aged less than 18 years.

Severity of the liver disease was assessed, for each patient, by estimating two clinical used clinical indices,i.e., the AST to Platelet Ratio Index (APRI = ((AST(IU/L)/ULN^*^) × 100)/((platelet counts (10^9^/L)) [4] and the Fibrosis-4 index (FIB-4 = (Age (years) × AST(IU/L))/(platelet count (10^9^/L) × √(ALT(IU/L)) [5].

### Laboratory procedures

For the measurement of leptin and vitamin D serum levels, serum samples were centrifuged at 2000 g/min for 15 min and stored at −20°C until their use.The assessment of these biomarkers was carried out at the laboratory of Clinical Biochemistry at the Gastrointestinal Tract Center of the Mansoura University, using ELISA assays (leptin kit from Bioassay Technology Laboratory, Shanghai, china; vitamin D kit from ImmunoCentric, Canga Park, USA). AST, ALT, platelet count, hemoglobin, WBCs, and RBCs were measured during the clinical routine. All the blood tests (leptin, vitamin D, and biochemical parameters) were performed in a single analytical session, following the instructions provided by the manufactures, and determination were performed by an operator without knowledge of the clinical information of the handled sample.

The following abbreviations were used for the remaining laboratory parameters. AST: aspartate aminotransferase; ALT: alanine aminotransferase; WBCs: white blood cells; RBCs: red blood cells.

### Shotgun proteome analysis

#### Extraction of serum proteins

Samples were precipitated with chilled acetone at −80 °C for 30 min, then at −20 °C overnight. Samples were centrifuged at 10,000 RPM for 30 min. The supernatant was discarded, and the tube was left at room temperature to evaporate the rest of the acetone. The pellet was reconstituted using 200 μl 8 M Urea (500 mM Tris pH 8.5) for protein denaturation. After that, samples were shaked vigorously and centrifuged on 10,000 RPM for 30 min at 4 ºC. Extracted proteins were collected and assayed using Bradford assay deNovix DS-11 FX Series Spectrophotometers and fluorometers at Å595 nm for quantification.

#### Protein lysate tryptic digestion

Thirty μg protein extract was reduced and alkylated with 2 μl of 200 mM Dithiothreitol (DTT) and 2 μl of 1 M Iodoacetamide (IAA), respectively. Samples were then diluted with 100 mM Tris pH 8.5 to reach around 2 M urea before trypsinization. 6μl trypsin containing 1ug porcine enzyme (Sigma, Germany) was added for endopeptidase digestion and incubated overnight at 37 °C with shaking at 900 rpm. The digested peptide solution was acidified using100% formic acid to a final pH of 2–3 [6]. Peptide mixture was cleaned up, and concentrated using reversed-phase Stage Tips (Pierce™ C18 Spin Tips; prod# 5010–21701). To condition C_18_ cartilage, 20 μl methanol was added and spin down at 1,500 g/1 min. The cartilage was rinsed with 50 μl 0.2% fluoroacetic acid (TFA), 80% acetonitrile (ACN) mixture and centrifuged at 1,500 g/1 min. Later on, 20 μl of 0.2% FA was applied on the disk and spin down again at 1,500 g/1 min. The eppendorf tube changed, and the sample added for sample trapping. C18 cartilage was washed with 20 μl of 0.2% FA. For Elution, in a collection tube recover 3 times 50μl from (0.2% FA + 80% ACN), then Speed-vac and re-constitute in 20 µl from FA. Samples were subjected to peptide quantification. The resultant peptide mixture was quantified using the BCA method (Pierce, Rockford IL) at Å562 nm.

#### Nano-LC MS/MS analysis

The study used a Nano-LC MS/MS analysis with an Eksigent nanoLC 400 autosampler and Ekspert nanoLC 425 pump connected to a TripleTOF 5600 + mass spectrometer. Peptides were trapped on a CHROMXP C18CL column and eluted with a linear gradient solution. The scan ranges were 400–1250 m/z and 170–1500 m/z, with collision-induced dissociation as the fragmentation source. Calibration was performed using PepcalMix every six hours.

#### Proteomics data analysis

Mascot generic format files were created from raw files using AB Sciex's script. MS/MS spectra were analyzed using X Tandem in Peptide Shaker against UniProt Homosapiens (a Swiss-Prot and TrEMBL database comprising 229,959 proteins) with target and decoy sequences (downloaded on 8.3.2021) [12].Precursor and fragment mass were identified, with a 1% false discovery rate. Carbamidomethylation, oxidation, acetylation, and variable modification were considered. Sample outputs were generated and normalized using probabilistic quotient normalization (PQN) [13].

### Gene ontology (GO) and Kyoto Encyclopedia of Genes and Genomes Functional Analysis (KEGG)

Gene ontology (GO) is a systematical approach for gene annotation, RNA and protein expression. KEGG is an online database of genomes, enzymatic pathways, and biochemicals. The pathway database of KEGG records molecular interaction networks in cells and changes specific to specific organisms [7].The Database for Annotation, Visualization and Integrated Discovery (DAVID) provides a comprehensive set of efficient and concise annotation tools for researchers to understand the biological meaning behind numerous genes [8]. In the present study,74 proteins were identified in the serum of liver fibrosis patients from proteomic analysis. GO and KEGG pathway enrichment analysis were used for proteins using the DAVID database. FDR < 0.05 was set as the cut-off criterion for the two analyses.

### Protein–Protein Interaction Analysis (PPI)

The proteins are in a homogenous environment inside the cell, performing multiple biological processes [9]. PPI networks can provide information on the molecular mechanism underlying cellular activity [10].The Search Tool for the Retrieval of Interacting Genes (STRING; http://string.embl.de/) is a biological database designed to construct a PPI network based on the known and predicted PPIs, and then analyze the functional interactions between proteins [14].In the present study, there are extracellular matrix proteins among proteins identified in the serum of liver fibrosis patients from proteomic analysis. These extracellular matrix proteins, besides leptin and vitamin D, were analyzed for protein–protein interaction (ppi) network using the STRING (version 12.0) database.

### Statistical analysis

Data are expressed as means ± SD. One-way ANOVA was used for analysis of leptin, vitamin D, APRI index values. These statistical analyses were performed using GraphPad Prism 8.0.2. Diagnostic accuracy of each hepatic fibrosis marker was assessed based on the area underneath the receiver operating characteristic curve (AUROC). The sensitivity, specificity, positive predictive value, negative predictive value and AUROC were determined for each hepatic fibrosis marker. Receiver operating characteristic curves were analyzed statistically using Medcalc 22.021.0. P ≤ 0.05 was considered significant.

## Results

### Comparative analysis of fibrosis biomarkers and fibrosis indices before and after the liver transplantation

**Fig. 1:**
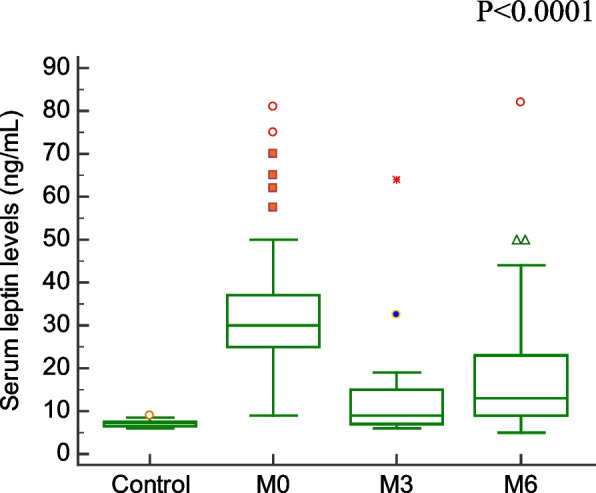
Serum leptin levels among the studied groups

**Fig. 2:**
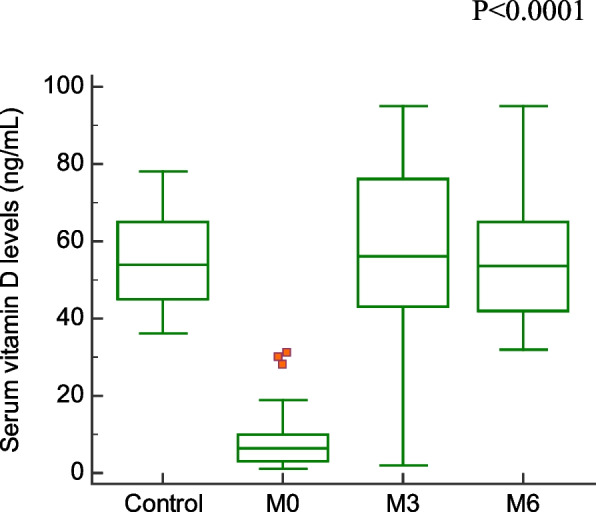
Serum vitamin D levels among the studied groups

**Fig. 3:**
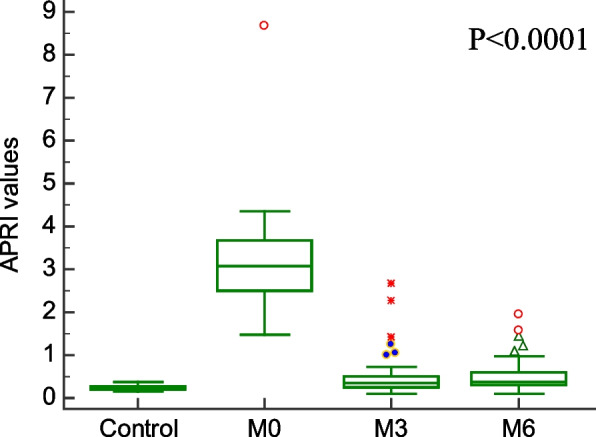
APRI values among the studied groups

**Fig. 4:**
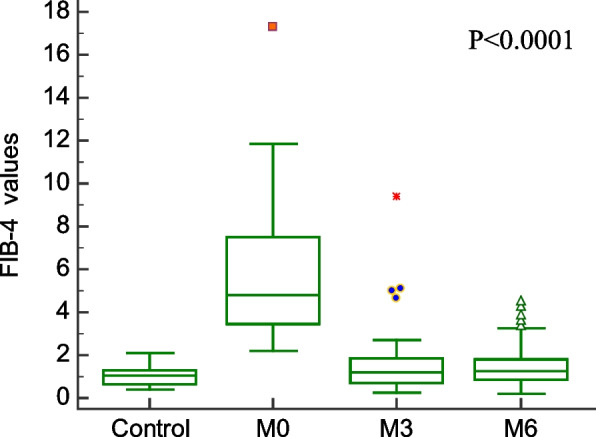
FIB-4 values among the studied groups

Comparison of demographic, clinical characteristics, and biopsy results of the studied groups are summarized in Table 1. In this section, We showed the corresponding comparative analysis. Serum concentrations of leptin were decreased from 33.89 ± 15.34 ng/mL at Pre-LT (M0) to 12.22 ± 8.974 ng/mL at three months post-LT (M3) and 18.25 ± 14.22 ng/mL at six months post-LT(M6) (*P* < 0.0001) (Fig. 1). Whereas vitamin D levels were increased from 7.720 ± 6.848 ng/mL at M0 to 56.00 ± 24.62 ng/mL at M3 and 55.00 ± 15.46 ng/mL at M6 (*P* < 0.0001) (Fig. 2). When APRI values were calculated, APRI values were decreased from 3.122 ± 1.535 at M0 to 0.5436 ± 0.5399 at M3 and 0.5384 ± 0.4209 at M6 (*P* < 0.0001) (Fig. 3). Where as FIB-4 values were decreased from 5.755 ± 2.965 at M0 to 1.580 ± 1.564 at M3 and 1.540 ± 1.069 at M6 (*P* < 0.0001) (Table 2) (Fig. 4). Serum concentrations of AST and ALT were decreased from 84.53 ± 19.85 IU/L, 83.55 ± 18.15 IU/L at Pre-LT (M0) to 28.14 ± 22.32 IU/L, 29.62 ± 20.60 IU/L at three months post-LT (M3) and 28.90 ± 21.58 IU/L, 28.90 ± 21.58 IU/L at six months post-LT(M6), respectively (*P* < 0.0001). Where as platelet count and hemoglobin concentration were increased from 70.85 ± 13.38 10^9^/L, 6.442 ± 1.298 gm % at Pre-LT (M0) to 182.9 ± 86.45 10^9^/L, 10.78 ± 1.787 gm % at three months post-LT (M3) and 177.5 ± 88.60 10^9^/L, 11.09 ± 1.660 gm % at six months post-LT(M6), respectively (*P* < 0.0001). WBCs and RBCs counts were increased from 2.976 ± 0.7032 10^9^/L, 2.954 ± 0.3477 10^9^/L at Pre-LT (M0) to 5.546 ± 2.050 10^9^/L, 4.442 ± 0.9457 10^9^/L at three months post-LT (M3) and 6.116 ± 2.235 10^9^/L, 4.708 ± 0.8554 10^9^/L at six months post-LT(M6), respectively (*P* < 0.0001). The results of comparative analysis of fibrosis markers and fibrosis indices indicate the potential effectiveness of the mentioned markers and indices for disease monitoring.

**Table 1:** Demographic, clinical characteristics and Biospy results of the studied groups

	Control	M0	M3	M6
Age (years)	44.03 ± 12.52	41.56 ± 13.71		
Gender (M/F)	15/15(50/50)	35/15(70/30)		
AST(IU/L)	21.91 ± 4.280	84.53 ± 19.85	28.14 ± 22.32	28.90 ± 21.58
ALT(IU/L)	18.18 ± 6.905	83.55 ± 18.15	29.62 ± 20.60	28.76 ± 18.60
Platelet count (10^9^/L)	230.4 ± 58.67	70.85 ± 13.38	182.9 ± 86.45	177.5 ± 88.60
Hemoglobin (gm %)	14.95 ± 1.991	6.442 ± 1.298	10.78 ± 1.787	11.09 ± 1.660
WBCs (10^9^/L)	7.943 ± 1.567	2.976 ± 0.7032	5.546 ± 2.050	6.116 ± 2.235
RBCs (10^9^/L)	4.500 ± 0.4550	2.954 ± 0.3477	4.442 ± 0.9457	4.708 ± 0.8554
Biopsy results			No. of events (%)	
Cirrhosis with steatosis 0%		18 (36%)		
Cirrhosis with steatosis 10%		4 (8%)		
Cirrhosis with steatosis 20%		2 (4%)		
Cirrhosis with steatosis 30%		1 (2%)		
Cirrhosis with steatosis 60%		5 (10%)		
Cirrhosis with Grade I HCC		11 (22%)		
Cirrhosis with Grade II HCC		7 (14%)		
Cirrhosis with Grade III HCC		2 (4%)		

Data are expressed as mean ± Standard deviation

**Table 2:** Values of Vitamin D, Leptin, APRI, and FIB-4

	Control	M0	M3	M6
Vitamin D	55.20 ± 11.53	7.720 ± 6.848	56.00 ± 24.62	55.00 ± 15.46
Leptin	7.130 ± 0.7502	33.89 ± 15.34	12.22 ± 8.974	18.25 ± 14.22
APRI	0.2503 ± 0.06708	3.098 ± 1.062	0.4980 ± 0.4925	0.4984 ± 0.3860
FIB-4	1.047 ± 0.4421	5.755 ± 2.965	1.580 ± 1.564	1.540 ± 1.069

Data are expressed as mean ± standard deviation

### Clinical significance of liver fibrosis biomarkers and indices in the progression of liver fibrosis: a comparison between cirrhotic patients with steatosis and cirrhotic patients with hepatocellular carcinoma

**Fig. 5:**
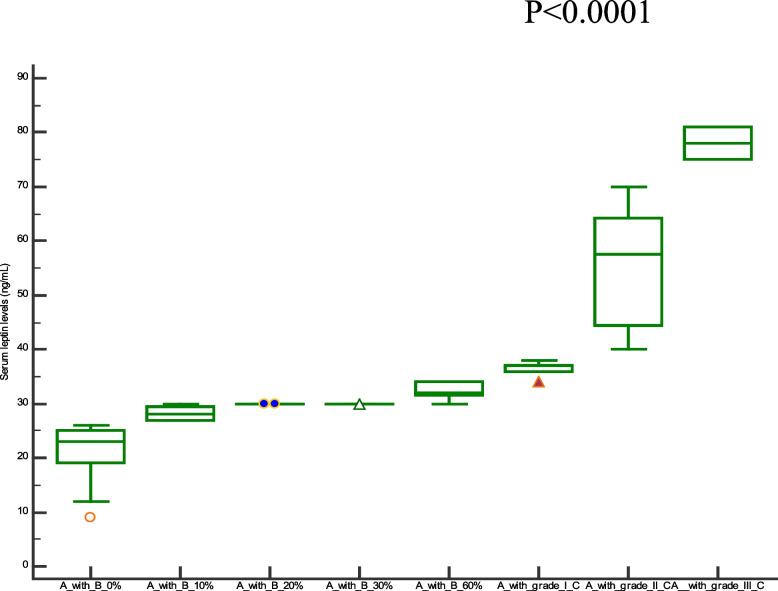
Serum leptin levels among cirrhotic patients with different ratios of Steatosis and cirrhotic patients with different grades of HCC. Where A = cirrhosis, B = steatosis, and C = Hepatocellular carcinoma

**Fig. 6:**
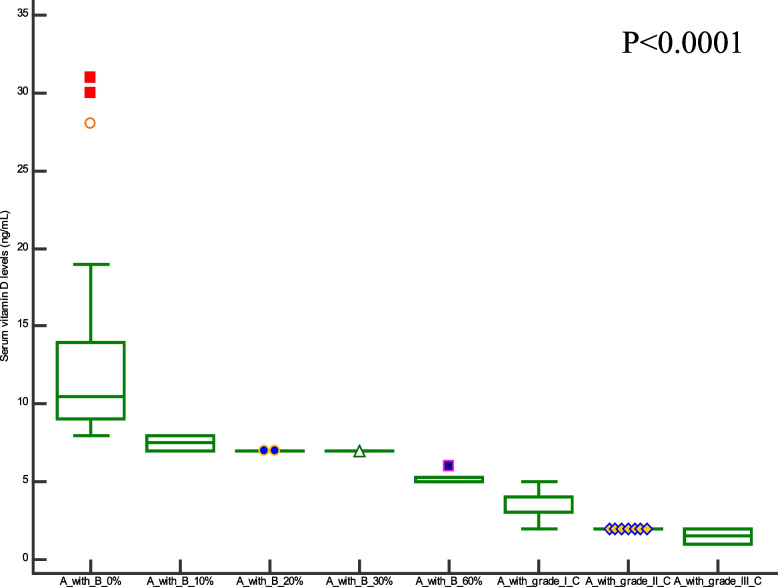
Serum vitamin D levels among cirrhotic patients with different ratios of Steatosis and cirrhotic patients with different grades of HCC. Where A = cirrhosis, B = steatosis, and C = Hepatocellular carcinoma

**Fig. 7:**
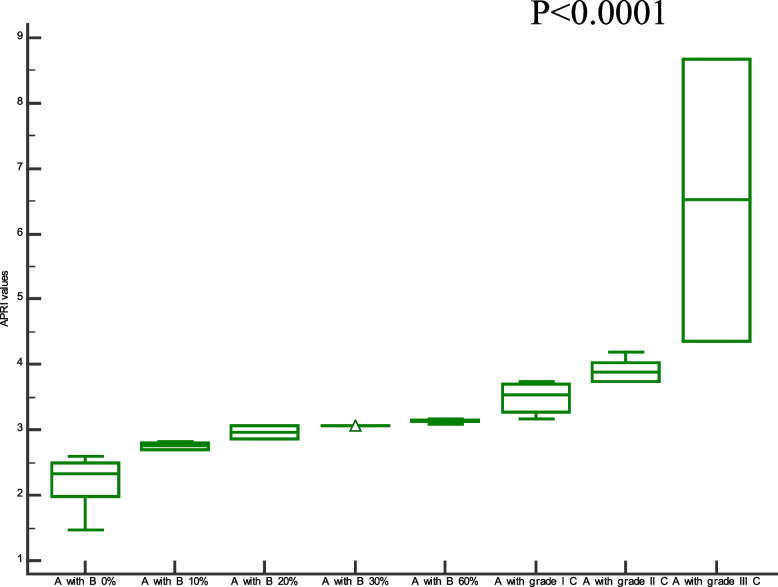
APRI values among cirrhotic patients with different ratios of Steatosis and cirrhotic patients with different grades of HCC. Where A = cirrhosis, B = steatosis, and C = Hepatocellular

**Fig. 8:**
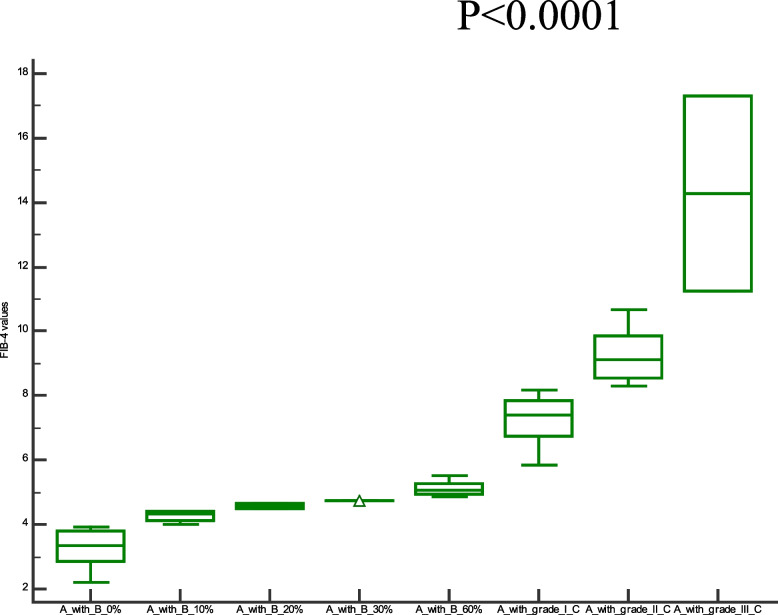
FIB-4 values among cirrhotic patients with different ratios of Steatosis and cirrhotic patients with different grades of HCC. Where A = cirrhosis, B = steatosis, and C = Hepatocellular carcinoma

To assess the clinical significant of the liver fibrosis markers and indices in liver carcinogenesis progression, in this section, we compare the values of selected liver fibrosis markers and indices in a cohort of 30 cirrhotic patients with different ratios of steatosis without any evidence of HCC and 20 cirrhotic patients with HCC. Data for leptin, vitamin D, APRI, and FIB-4 values are visualized in Figs. 5, 6, 7 and 8. There has been a statistically significant increase in vitamin D levels in patients with different grades of HCC, while leptin, APRI, and FIB-4 values have decreased in patients with different grades of HCC((*P* < 0.0001).

### Analysis of liver fibrosis biomarkers in patients and correlation with clinical parameters

**Fig. 9:**
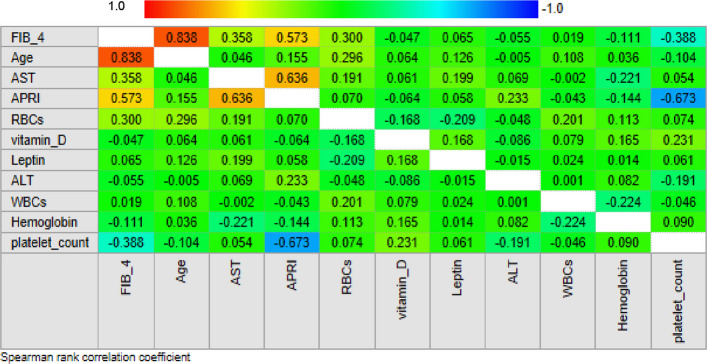
Correlation matrix of Spearman's coefficients, ρ, between the investigated fibrosis markers and a panel of selected plasma biochemical parameters and clinical indices for fibrosis (APRI and FIB-4). The coloring scale is used to assess the direction of the correlations, with strong negative values displayed in blue and positive ones in light red.The strength of the significant correlation is directly proportional to the pixel size and the pixel intensity. Non-significant correlations are indicated as empty white pixels

In this section,we describe the investigation of the clinical significance of selected serum liver fibriosis biomarkers (leptin and vitamin D) in a cohort of pateints for disease monitoring. The possible use of liver fibrosis markers for diagnostic purposes is investigated in Fig. 9, in which we study the correlation between these biomarkers and two widely used clinical indices, namely APRI and FIB-4 indices.

Specificially, Fig. 9 shows a correlation matrix of Spearman's coefficients (ρ) computed between the investigated fibrosis markers and the two mentioned fibrosis indices. The ρ values for a panel of conventional biochemical parameters are also included in the analysis. This Figure shows the prescence of several significant correlations between APRI values and FIB-4 (*ρ* = 0.573, *p* = 1.365e-005), AST (*ρ* = 0.635, p = 7.088e-007), and platelet count (*ρ* = −0.672, *p* = 8.834e-008) and between FIB-4 values and AST (*ρ* = 0.358, *p* = 0.0106), platelet count (*ρ* = −0.388, p = 0.005), age (*ρ* = 0.838, p = 3.169e-014), and RBCs (*ρ* = 0.3004, *p* = 0.0106). No significant correlation with leptin and vitamin D.

### Diagnostic performance of serum leptin and vitamin d in predicting liver fibrosis

**Fig. 10:**
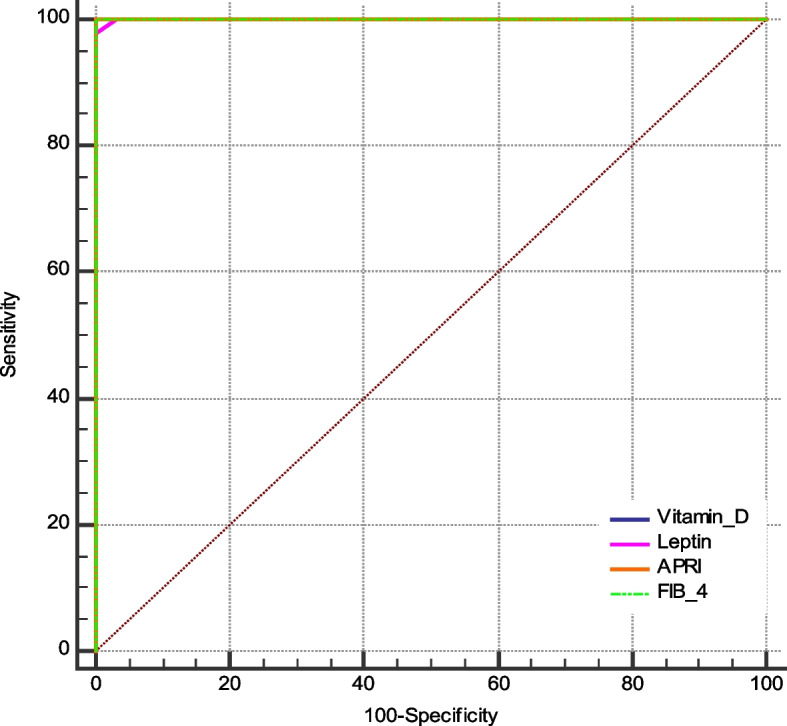
The ROC curves comparing the performance of serum leptin and vitamin D levels with APRI and FIB-4 indices using liver biopsy as the gold standard

We performed ROC Curve to discriminate between between healthy and liver fibrosis pateints before going to liver transplantion (Pre-LT) using liver biospy as the gold standard. As shown in Fig. 10, the AUROC of the four indices (leptin, vitamin D, APRI and FIB-4) was 1, which indicate perfect discrimination in predicting liver fibrosis. Take both sensitivity and specificity into account, the cut-off point was selected according to max number of sensitivity and specificity. Table 3 showed the cut-off point, sensitivity, specificity, area under the curve (AUC), P-value, positive predictive value (+ PV), and negative predictive value(-PV) of the four indices.

**Table 3:** ROC curves of four serum fibrosis indices

Indices	AUC	*P*-value	cut-off value	Sensitivity	Specificity	+ PV	-PV
Vitamin D	1	< 0.0001	≤ 31	100%	100%	100%	100%
Leptin	1	< 0.0001	> 9	98%	100%	100%	96.8%
APRI	1	< 0.0001	> 0.38	100%	100%	100%	100%
FIB-4	1	< 0.0001	> 2.08	100%	100%	100%	100%

### Qualitative proteomic analysis

Data matrices of protein intensities were obtained by preprocessing LC–MS/MS runs based on ion intensity and spectral count. Only Proteins with more than two identified peptides were considered. In the MaxQuant analysis 74 proteins were identified in serum of liver fibrosis patients. The complete lists of the identified proteins are provided in Table 4.

**Table 4:** Proteins identified by qualitative proteomic analysis

Protein name	Uniprot ID	Protein name	Uniprot ID
Apolipoprotein A-I	P02647	Complement factor B	P00751
Apolipoprotein A-II	P02648	Complement factor H	P08603
Apolipoprotein A-IV	P02649	Complement C1r subcomponent	P00736
Apolipoprotein B-100	P02650	Complement C1s subcomponent	P09871
Apolipoprotein C-I	P02651	Complement component C7	P10643
Apolipoprotein C-II	P02652	Complement component C9	P02748
Apolipoprotein C-III	P02653	Complement C1q subcomponent subunit B	P02746
Apolipoprotein E	P02654	Complement C1q subcomponent subunit C	P02747
Apolipoprotein M	P02655	Ceruloplasmin	P00450
Alpha-1-acid glycoprotein 1	P02656	Clusterin	P10909
Alpha-1-acid glycoprotein 2	P02657	CD5 antigen-like	O43866
Alpha-1-antichymotrypsin	P02658	Carboxypeptidase N subunit 2	P22792
Alpha-1-antitrypsin	P02659	C-reactive protein	P02741
Alpha-1B-glycoprotein	P02660	Fibronectin	P02751
Alpha-2-macroglobulin	P02661	Gelsolin	P06396
Alpha-2-antiplasmin	P02662	Haptoglobin	P00738
Alpha-2-HS-glycoprotein	P02663	Hemopexin	P02790
Inter-alpha-trypsin inhibitor heavy chain H1	P02664	Heparin cofactor 2	P05546
Inter-alpha-trypsin inhibitor heavy chain H2	P02665	Histidine-rich glycoprotein	P04196
Inter-alpha-trypsin inhibitor heavy chain H4	P02666	Hemoglobin subunit alpha	P69905
Beta-2-glycoprotein 1	P02667	Hemoglobin subunit beta	P68871
Albumin	P02668	Immunoglobulin kappa variable 2–40	A0A087WW87
Afamin	P02669	Immunoglobulin lambda-like polypeptide 1	P15814
Angiotensinogen	P02670	Immunoglobulin lambda-like polypeptide 5	B9A064
ATP-binding cassette sub-family F member 1	P02671	Immunoglobulin J chain	P01591
Antithrombin-III	P02672	Kininogen-1	P01042
Prothrombin	P02673	Lymphocyte cytosolic protein 2	Q13094
Plasminogen	P02674	Lipopolysaccharide-binding protein	P18428
Platelet basic protein	P02675	Leucine-rich alpha-2-glycoprotein	P02750
Plasma protease C1 inhibitor	P02676	Lumican	P51884
Plasma kallikrein	P02677	Maestro heat-like repeat-containing protein family member 2B	Q7Z745
Protein AMBP	P02678	Serotransferrin	P02787
Pigment epithelium-derived factor	P02679	SHC-transforming protein 1	P29353
Complement C3	P02680	Transthyretin	P02766
Complement C5	P02681	Vitamin D-binding protein	P02774
Complement C4-A	P02682	von Willebrand factor	P04275
Complement factor I	P02683	Vitronectin	P04004

### Gene ontology analysis

**Fig. 11:**
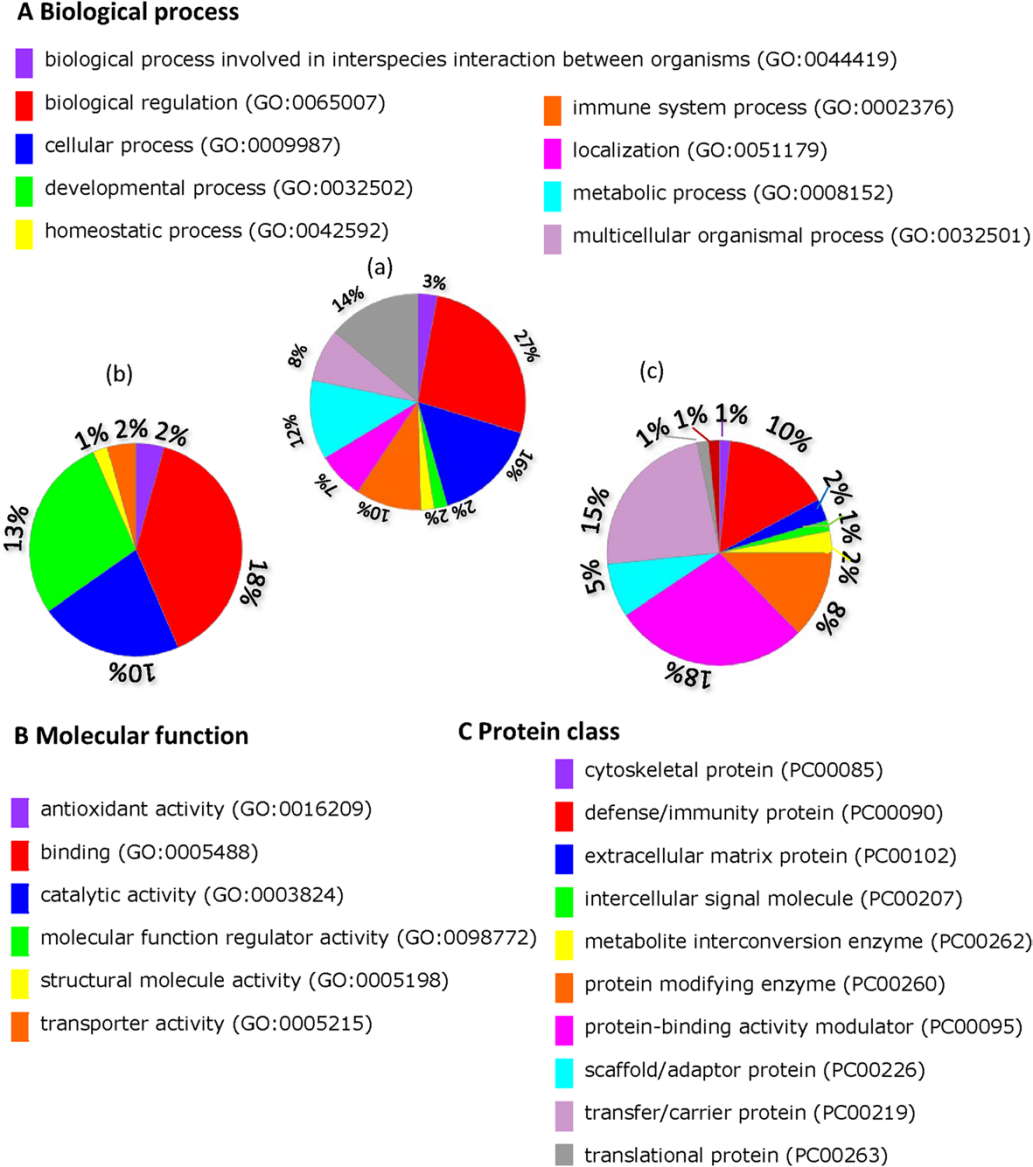
Go classifications of the 74 proteins using the PANTHER database. The obtained 74 proteins were classified into three GO categories: biological process (**a**), molecular function (**b**), and protein class (c). Displayed percentages are based on protein numbers for a given GO category

Gene ontology analysis was performed using PANTHER [15] based on the 74 proteins identified in serum of liver fibrosis patients (Fig. 11).

### Kyoto Encyclopedia of Genes and Genomes Functional Analysis (KEGG) pathway analysis

**Fig. 12:**
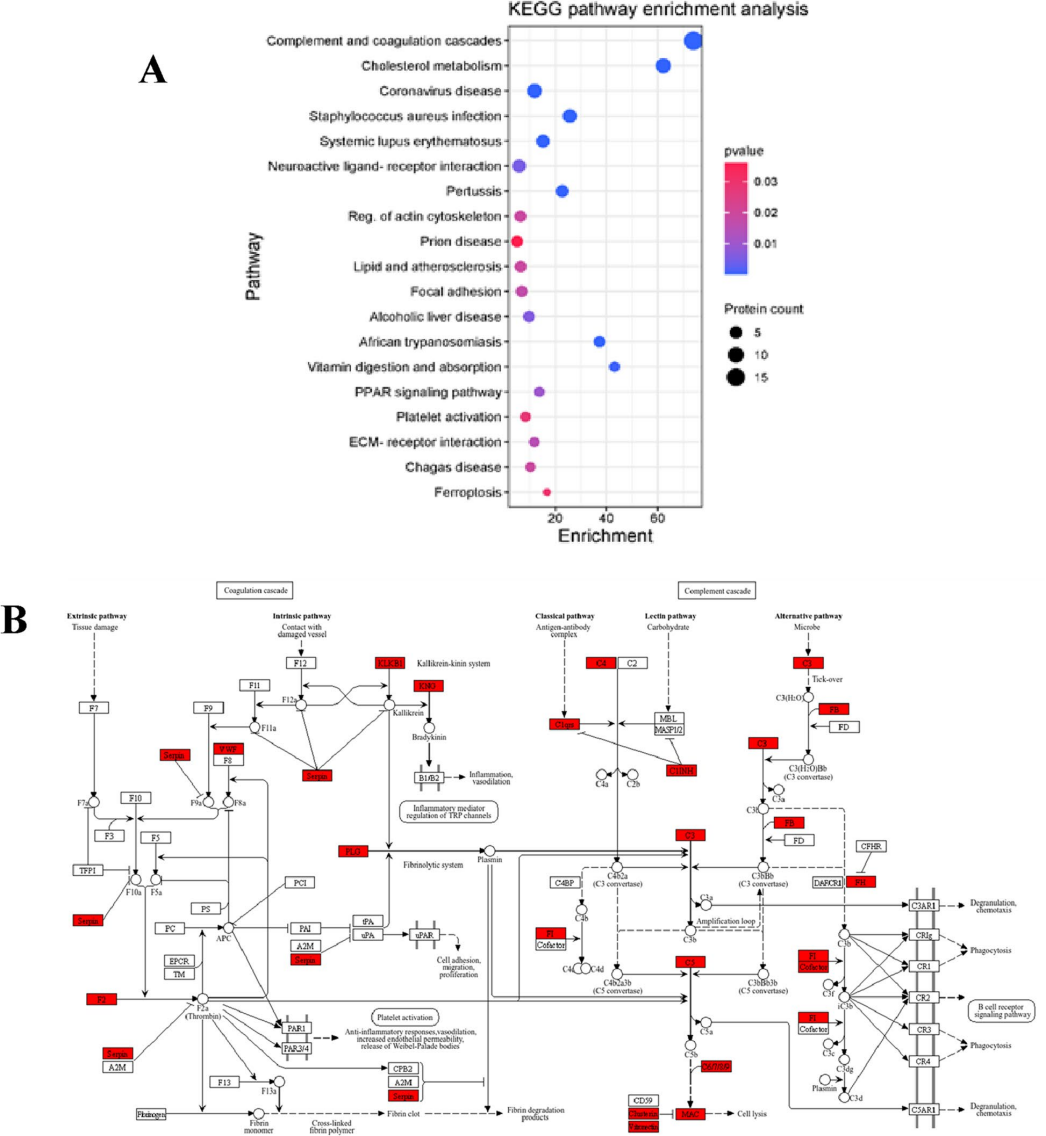
KEGG pathway enrichment analysis

KEGG pathway analysis of proteins differentially expressed in serum of liver fibrosis patients showed significant enrichment of 19 pathways (Fig. 12 A (. Among the enriched pathways, complement and coagulation cascades (Fig. 12 B).

### Protein- protein interaction (PPI) network

**Fig. 13:**
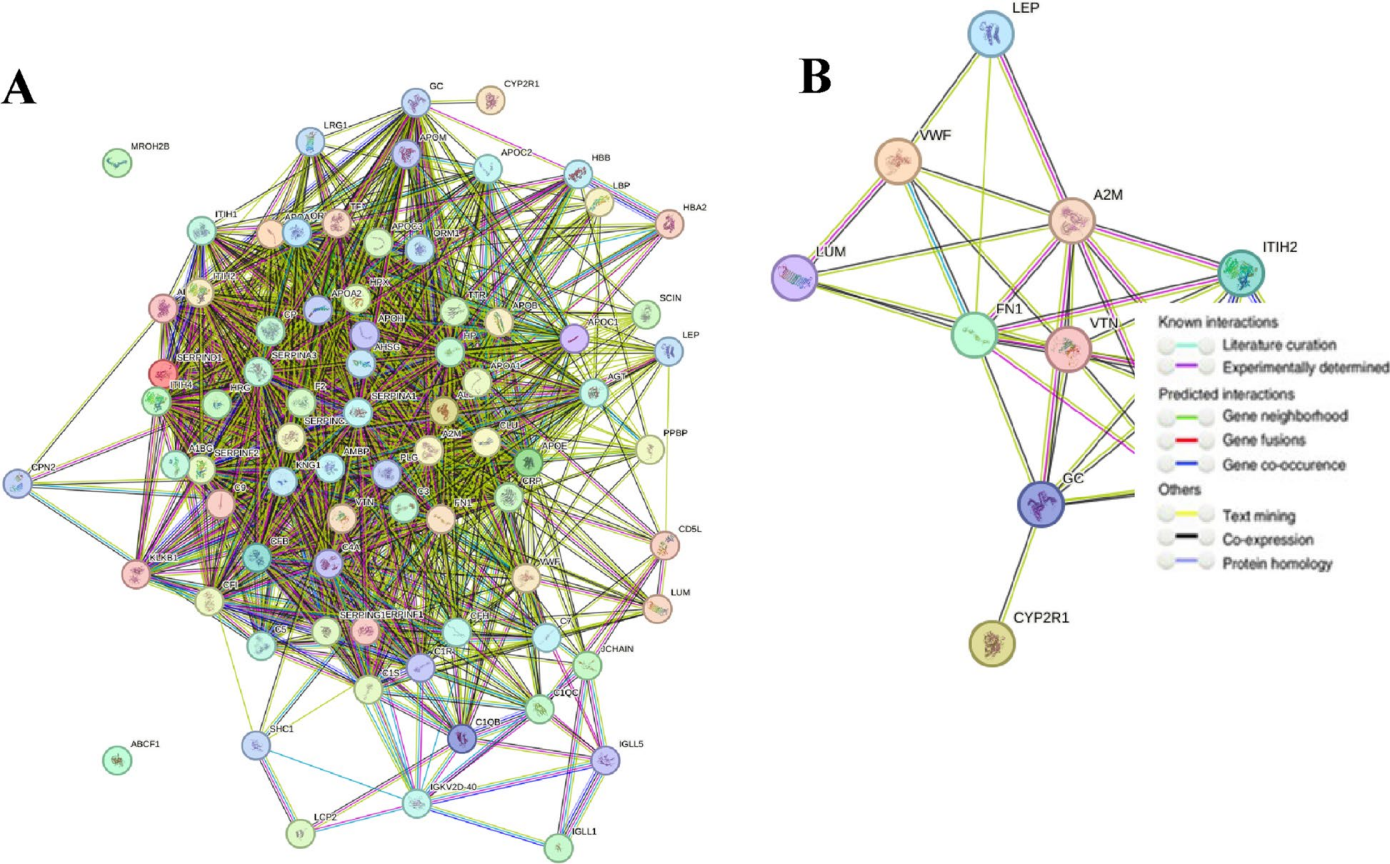
PPI network obtained from STRING online database. The nodes in the network are proteins and the edges are protein–protein interaction. The sources of interactions between nodes in PPI network are shown by various colors. The protein–protein interaction (PPI) network of leptin and vitamin D with the total proteins identified in the serum of liver fibrosis patients (Fig. 13 **A**), while the PPI network of leptin and vitamin D with extracellular matrix proteins identified in the serum (Fig. 13 **B**)

The protein–protein interaction (PPI) network of leptin and vitamin D with the total proteins identified in the serum of liver fibrosis patients (Fig. 13 A), while the PPI network of leptin and vitamin D with extracellular matrix proteins identified in the serum (Fig. 13 B), and the PPI network was constructed using the string database was constructed using the string database. In the string database, the association between queries are derived from high-throught experimental data and computational predictions. the network statistics for network of leptin and vitamin D with the total proteins showed the total number of nodes(*n* = 76), edges (*n* = 1266) and the expected number of edges (*n* = 77). The average node degree or average number of interactions exhibited by a protein was 33.3, with average local clustering coefficient of 0.717. The PPI enrichment p-value (< 1.0e-16) was significant, while the network statistics for network of leptin and vitamin D with extracellular matrix proteins showed the total number of nodes(*n* = 12), edges (*n* = 37) and the expected number of edges (*n* = 3). The average node degree or average number of interactions exhibited by a protein was 6.17, with average local clustering coefficient of 0.84. The PPI enrichment p-value (< 1.0e-16) was significant.

## Discussion

Leptin regulates a number of basic cellular and molecular biology processes involved in the development and resolution of hepatic fibrosis [12]. HSCs have been shown to produce leptin when they get activated. These findings lead to the hypothesis that leptin plays a pivotal role in profibrogenic responses in the liver. Leptin possesses profibrogenic and proinflammatory properties [13]. During the regression of liver fibrosis, the number of activated hepatic stellate cells is greatly reduced by the induction of cellular senescence and apoptosis, or by the return to the quiescent state[16].

In our study we found that serum leptin levels were significantly higher in liver fibrosis patients than in controls. Piche et al., also stated that mean fasting serum leptin levels were [17] significantly higher in patients than in controls [18]. After 3- and 6-months following LT serum leptin significantly decreased. Such a result is in accordance with the study obtained by Roberts et al., that showed that serum leptin fell to levels which were significantly lower than pre-OLT values [19].

Higher levels of serum leptin at pre-LT showed that leptin possessed profibrogenic and proinflammatory properties, which only the result of the activation of HSCs in the liver. Lower levels of serum leptin at 3- and 6-months post-LT showed that successful LT affects the liver through regression of liver fibrosis and conversion of the HSCs from the activated state into a quiescent state that does not produce leptin.

Vitamin D has anti-proliferative and anti-fibrotic properties in HSCs by suppressing the nuclear translocation of mothers against the decapentaplegic homolog signaling pathway and blocking the activity of pro-fibrotic TGF-β [20].

This study showed that serum vitamin D concentrations significantly decreased in liver fibrosis patients than control. Such a result is in accordance with the study obtained by Malham et al. that showed that serum vitamin D levels decreased in liver disease [17]. After 3- and 6-months following LT serum vitamin D significantly increased.

Decreased serum levels of vitamin D at pre-LT showed that liver disease decreased vitamin D hydroxylation, which is only partly the result of a synthesis dysfunction of the liver. Increased serum levels of vitamin D at 3- and 6-months post-LT showed successful LT-affected vitamin D serum levels through which vitamin D played a role as an anti-fibrotic marker.

Over the past ten years, non-invasive laboratory and radiological tests have been developed as preferred methods to evaluate liver fibrosis in PPHNA, and they are expected to overcome the limitations of liver biopsy. Fibrosis-4 (FIB-4), the AST to Platelet Ratio Index (APRI), and the Aspartate Aminotransferase (AST)/Alanine Aminotransferase (ALT) Ratio comprise this technique [21].

Fibrosis index based on four factors (FIB-4) was first developed to access liver fibrosis comprised of age, aspartate aminotransferase (AST), alanine aminotransferase (ALT), and platelet [22].APRI, or the Aspartate Aminotransferase Platelet Ratio Index, is a simple, easily accessible, repeatable, affordable, and accurate non-invasive diagnostic tool for hepatic fibrosis. It was reported that APRI is a novel index for predicting severe cirrhosis and fibrosis [21]. Since AST and platelet count are used as indicators in the formulation of the APRI score, an increase in AST levels and a reduction in platelet count are indicative of a more severe case of liver fibrosis. The release of AST from mitochondria and the reduction in AST clearance resulting from liver fibrosis are linked to an increase in AST levels and hepatocyte damage. Decreased thrombopoietin production by injured hepatocytes is the cause of the decrease in platelet numbers [23].

In our study we found that the APRI and FIB-4 values were significantly higher in liver fibrosis patients than in controls. After 3- and 6-months following LT, APRI values significantly decreased. Such a result is consistent with the study obtained by Mansoor et al. that showed that the APRI and FIB-4 were significantly higher in patients with significant fibrosis than with no fibrosis [24].

Notably,cirrhotic patients with different grades (I, II, and III) of HCC show higher values of leptin,vitamin D, FIB-4, and APRI than cirrhotic pateints with different grades of steatosis (0, 10, 20, 30, 60%). These values in cirrhotic pateints with grade III higher than in grade I and II HCC, Also with cirrhotic pateints with 60% steatosis higher than in 30%, 20%, 10%, and 0%.This indicates that leptin,vitamin D, FIB-4, and APRI play a role in the tumor development and progression.

The FIB-4 index has been found to significantly correlate positively with AST, age, and RBCs and negatively with platelet count, and APRI has also been found to correlate positively with AST and FIB-4 and negatively with platelet count, and its identification can help in estimating the severity of disease. A more severe degree of liver fibrosis resulted from a higher level of each marker. This supported the earlier theory that the degree of liver fibrosis was directly correlated with the increase in each marker level. AST and platelets are used as indicators in the FIB-4 index and APRI score formulation; hence, an increase in AST levels and a drop in platelet count correspond to the severity of liver fibrosis. A rise in AST levels is linked to hepatocyte injury, which causes AST to be released from mitochondria and reduces AST clearance because of liver fibrosis. Reduced thrombopoietin production by injured hepatocytes is the cause of the drop in platelet numbers [23].

AUC and ROC curves can be used to evaluate the efficacy of a single test.

ROC curves can compare many diagnostic methods for a single illness. Clinicians can receive assistance in identifying the best plan in this way. ROC analysis is now a crucial technique for evaluating hepatic fibrosis diagnostic indicators [25]. In this study, we compared the diagnostic performances of serum leptin and vitamin D in predicting liver fibrosis. The results showed that the AUROC of the four indices (leptin, vitamin D, APRI and FIB-4) was 1, which indicate perfect discrimination in predicting liver fibrosis.The cut-off point is used in our study to confirm the critical value of serum fibrosis indices when they are used in the diagnosis of hepatic fibrosis.

To prove also that leptin and vitamin D are good biomarkers for diagnosing liver fibrosis, we first identified the total proteins using proteomics analysis, then identified the role of these proteins in liver fibrosis pathogenesis using GO and KEGG analysis, later we identified the relationship between leptin and vitamin D and the proteins identified through STRING database.

We performed qualitative proteomic analysis to identify the proteins in the sera from patients with liver fibrosis. Among these proteins, there are extracellular matrix proteins.

To illustrate the biological processes and pathway that all proteins were involved, we used gene ontology analysis and KEGG pathway enrichment analysis respectively.

Gene ontology analysis demonstrated that the proteins participate in a diverse array of biological processes, including metabolic process, cellular process, developmental process, immune system process, homeostatic process, localization, etc., emphasizing the systemic effects of liver fibrosis disease. Gene ontology analysis also reveals that the majority of these proteins were defense/immunity protein.

In the present study, KEGG pathway enrichment analysis demonstrated that these proteins were predominantly enriched in the complement and coagulation cascades. Along with complement and coagulation cascades, ECM-receptor interaction and PPAR signaling pathway have been confirmed to serve a critical role in liver fibrosis development. The innate and adaptive immune systems are both impacted by the network of soluble serum proteins, membrane-bound receptors, and regulatory proteins that make up the complement system. The liver is where complement activation primarily takes place [26]. It has been demonstrated that the coagulation cascade contributes to fibrosis and chronic liver damage [27].

After identifying the role of these proteins in the pathogenesis of liver fibrosis, the next procedure is to find the relationship between total proteins and extracellular matrix proteins with leptin and vitamin D.

Among the total proteins identified, there are extracellular matrix proteins. Liver fibrosis is a common outcome of chronic liver disease, characterized by accumulating extracellular matrix proteins [28] such as fibronectin [29], alpha-2-macroglobulin [30], von Willebrand factor [31], lumican [32], The inter-alpha-trypsin inhibitors (ITI) [33], etc. The Inter-alpha-trypsin inhibitors (ITI) are a family of plasma protease inhibitors that are composed of five homologous heavy chains (encoded by ITIH1, ITIH2, ITIH3, ITIH4, and ITIH5) and one light chain (bikaunin, encoded by AMBP). By covalently attaching to hyaluronan, the ITIH family of inhibitors contributes to the stability of the extracellular matrix [33].

In our study,serum leptin concentration was higher in liver fibrosis patients than in controls, while vitamin D concentration was lower than controls and previous studies have reported that vitronectin is increased in chronic inflammatory liver disease [34],The VWF levels were higher in patients with severe liver fibrosis stage and/or HCC development [31], Lumican is abundant in fibrotic tissues [35], and serum levels of ITIH4 were significantly correlated with higher stages of liver fibrosis indicating a possible relation to liver fibrogenesis [36].

To illustrate the interactions between leptin and vitamin D with total proteins and extracellular matrix proteins found in serum from liver fibrosis patients, we used the current STRING database.

From the STRING database, the proteins are at least partially biologically connected, as a group with leptin and vitamin D explaining the promiscuous nature of leptin and vitamin D. This emphasized the role of leptin and vitamin D in accumulation and increased synthesis of extracellular matrix proteins that incorporated in the pathogenesis of liver fibrosis, and showed that the leptin and vitamin D are involved in the pathogenesis process where the total proteins are involved.

Bioinformatics analysis provide valuable information to better understand the mechanisms of total proteins and the extracellular matrix proteins identified in serum of patients with liver fibrosis and their relation to leptin and vitamin D.

## Conclusion

In summary, serum vitamin D and leptin could serve as a valuable supplemental biomarker for predicting the liver fibrosis.

## Data Availability

No datasets were generated or analysed during the current study.

## Abbreviations

APRI: Aspartate aminotransferase to platelet count ratio
AUC: Area under the curve
STRING: The Search Tool for the Retrieval of Interacting Genes
TGF-β1: Transforming growth factor- β1
HSC: Hepatic stellate cells.
GO: Go Ontology.
KEGG: Kyoto Encyclopedia and Genomes Functional Analysis.
PPI: Protein–Protein Interactions
